# The Regulation of Ferroptosis by Tumor Suppressor p53 and its Pathway

**DOI:** 10.3390/ijms21218387

**Published:** 2020-11-09

**Authors:** Juan Liu, Cen Zhang, Jianming Wang, Wenwei Hu, Zhaohui Feng

**Affiliations:** Department of Radiation Oncology, Rutgers Cancer Institute of New Jersey, Rutgers-State University of New Jersey, New Brunswick, NJ 08903, USA; jl1530@cinj.rutgers.edu (J.L.); zhcen7917@gmail.com (C.Z.); jw1102@gsbs.rutgers.edu (J.W.)

**Keywords:** tumor suppressor, p53, ferroptosis, metabolism, lipid peroxidation, cancer, disease

## Abstract

Tumor suppressor p53 plays a key role in tumor suppression. In addition to tumor suppression, p53 is also involved in many other biological and pathological processes, such as immune response, maternal reproduction, tissue ischemia/reperfusion injuries and neurodegenerative diseases. While it has been widely accepted that the role of p53 in regulation of cell cycle arrest, senescence and apoptosis contributes greatly to the function of p53 in tumor suppression, emerging evidence has implicated that p53 also exerts its tumor suppressive function through regulation of many other cellular processes, such as metabolism, anti-oxidant defense and ferroptosis. Ferroptosis is a unique iron-dependent form of programmed cell death driven by lipid peroxidation in cells. Ferroptosis has been reported to be involved in cancer, tissue ischemia/reperfusion injuries and neurodegenerative diseases. Recent studies have shown that ferroptosis can be regulated by p53 and its signaling pathway as well as tumor-associated mutant p53. Interestingly, the regulation of ferroptosis by p53 appears to be highly context-dependent. In this review, we summarize recent advances in the regulation of ferroptosis by p53 and its signaling pathway. Further elucidation of the role and molecular mechanism of p53 in ferroptosis regulation will yield new therapeutic strategies for cancer and other diseases, including neurodegenerative diseases and tissue ischemia/reperfusion injuries.

## 1. Introduction

Tumor suppressor p53 plays a key role in tumor suppression [1,2,3,4]. p53 function is disrupted in many human cancers through mutations of the p53 gene and other mechanisms, including amplification and/or overexpression of p53 negative regulators (e.g., MDM2 and MDM4), which is a prerequisite for the initiation and/or progression of many human cancers [1,2,3,4,5]. The p53 gene is mutated in over half of all human cancers and almost in every type of cancers [6,7,8,9]. As a transcription factor, p53 mainly exerts its tumor suppressive function through selective transcriptional regulation of many target genes to regulate various fundamental cellular responses, including apoptosis, cell cycle arrest, senescence, DNA repair and metabolism [1,2,3,4]. In addition to transcription regulation, p53 also directly interacts with other proteins to regulate different cellular responses, such as apoptosis and DNA repair [10,11]. In human cancers, majority of p53 mutations are missense mutations, which leads to the production of full-length mutant p53 (mutp53) proteins in cancer cells [5,6,7,8]. Different from wild-type p53 proteins in normal cells, mutp53 proteins frequently accumulate to very high levels in cancer cells. Notably, many missense mutp53 proteins have been demonstrated to not only lose the tumor suppressive function of wild-type p53 but also exert gain-of-function (GOF) activities to promote cancer progression independently of wild-type p53 [5,6,7,8,12,13].

Recent studies have revealed a novel function of p53 in regulation of ferroptosis, a unique iron-dependent form of cell death driven by the accumulation of lipid-based reactive oxygen species (ROS) in cells [14,15,16,17,18]. Ferroptosis is a specific form of cell death that was originally found to be induced by small molecules erastin and RSL3 (RAS-selective lethal 3), which were identified in the synthetic lethal screening for small molecules targeting cancer cells with overexpression of oncogenic RAS [14,19,20]. Ferroptosis has been reported to be involved in different physiological and pathological processes, including cancer, neurodegenerative diseases, tissue ischemia/reperfusion injuries and immune response [15,16,17,18,21]. Recent studies reported that the regulation of ferroptosis by p53 contributes to the tumor suppressive function of p53 and furthermore, mutp53 protein accumulation in cancer cells sensitizes cancer cells to ferroptosis [22,23,24]. In this review, we summarize the current understanding of the role and mechanism of p53 and its signaling pathway in the regulation of ferroptosis as well as its potential impact upon the development and therapies of cancer and other diseases.

## 2. The p53 Signaling Pathway

**Figure 1:**
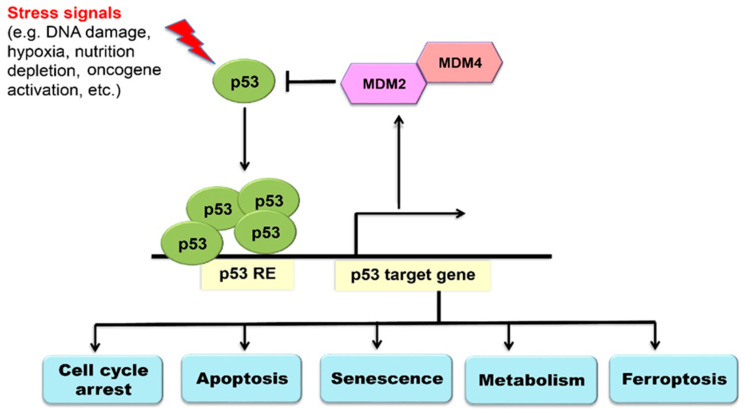
Tumor suppressor p53 signaling pathway. p53 protein is accumulated and activated in cells in response to various stress signals. Once activated, p53 transcriptionally regulates the expression of select target genes to regulate different cellular processes, including cell cycle arrest, senescence, apoptosis, metabolism and ferroptosis. MDM2 and MDM4 play a critical role in negative regulation of p53 in cells. p53 RE: p53-responsive element.

To ensure the proper function of p53 in regulating many fundamental cellular processes, p53 protein levels and activity are tightly regulated in cells. While p53 protein is usually maintained at low levels in normal cells under non-stressed conditions, the half-life of p53 protein is dramatically increased, leading to p53 protein accumulation in cells in response to a wide variety of intracellular and extracellular stress signals, such as DNA damage, hypoxia, nutrition depletion, activation of oncogenes and so forth. Once activated, p53 binds to the p53-responsive elements in its target genes to transcriptionally regulate their expression [1,2,3,4]. Through transcriptional regulation of select target genes in a highly cell- and tissue-type-specific and stress signal-specific manner, p53 regulates various cellular responses, including apoptosis, cell cycle arrest, senescence, DNA repair, metabolism, anti-oxidant defense, autophagy and ferroptosis, contributing to the role of p53 in tumor suppression [2,3,4,25,26] (Figure 1). In addition to tumor suppression, p53 has also been demonstrated to be involved in the regulation of many other biological and pathological processes, including anti-infection, immune response, maternal reproduction, development, tissue ischemia/reperfusion injuries, neurodegenerative diseases and aging [2,3,4,26,27,28,29,30,31,32]. Interestingly, recent studies have revealed that ferroptosis is also involved in tumor suppression, tissue ischemia/reperfusion injuries, neurodegeneration and immune response [15,16,17,21].

While p53 is regulated by many different mechanisms, the post-translational modifications, especially the ubiquitination modification, are the most critical and efficient mechanism to regulate the level and activity of p53 [4,33,34,35,36]. E3 ubiquitin ligase MDM2 is the most critical negative regulator of p53, which ubiquitinates and degrades p53 [37,38]. MDM2 is also a p53 target gene; p53 transcriptionally induces MDM2. Thus, MDM2 forms a negative feedback loop with p53 to tightly regulate p53 [37,38,39]. MDM4 (also named MDMX), a structural homology of MDM2, acts as another important negative regulator of p53 [37,38,40]. Although MDM4 lacks the E3 ubiquitin ligase activity, MDM4 forms a complex with MDM2 to stabilize MDM2 and promote MDM2-mediated ubiquitination and degradation of p53 [37,38,40]. Further, MDM4 inhibits p53 transactivation capacity through binding to p53 [37,39,40] (Figure 1). 

Interestingly, over 80% of p53 mutations in human cancers are missense mutations, leading to the production of full-length mutp53 proteins [5,6,7,8,9,12,13]. Further, ~90% of p53 mutations occur in the DNA-binding domain of p53, including several mutational hotspots (e.g., R175, R245, R248, R249, R273 and R282) that account for around one third of p53 mutations in cancers, which impairs the ability of p53 to bind to its target genes to transcriptionally regulate these genes [5,6,7,8,9,12,13]. Numerous studies have shown that many missense mutp53 proteins, including the hotspot mutp53, display GOF activities to promote cancer progression in the absence of wild-type p53 [5,6,7,8,12,13]. These GOF activities include promoting proliferation, metastasis, genomic instability, metabolic reprograming and therapeutic resistance of cancer cells [5,6,7,8,12,13]. Studies have also revealed that mutp53 can exert its GOF activities through many different mechanisms [5,6,7,8,12,13]. For instance, GOF mutp53 binds to different transcription factors and co-factors (e.g., p63/p73, NF-Y, VDR, ETS1/2, SREBPs, Sp1, etc.) and is recruited to their binding sites to affect the transcription of their target genes [5,6,7,8,12,13]. GOF mutp53 also interacts with select proteins in addition to transcription factors to regulate their levels, activities and/or functions to promote tumorigenesis [5,6,7,8,12,13]. 

## 3. The Signaling Pathways in Ferroptosis

Growing evidence have shown that ferroptosis is involved in different physiological and pathological processes, including tissue ischemic injury (e.g., ischemic heart diseases, brain damage, kidney failure and lung injury) [41,42,43,44] and neurodegeneration (e.g., Parkinson’s, Huntington’s and Alzheimer’s diseases) [45,46,47]. For instance, knockout of glutathione peroxidase 4 (GPX4), a key protein involved in ferroptosis regulation, in mice leads to ferroptosis and acute renal failure [41]. The intracerebral hemorrhage-induced ferroptosis contributes to neuronal death in mouse models [42]. Deletion of GPX4 in forebrain neurons in mice induces ferroptosis and leads to cognitive impairment and neurodegeneration [46]. Ferroptosis inhibitors, such as ferrostatins, liproxstatins and iron chelators, protect animals from ischemic injury in the liver, kidney, brain and heart [41,43,47,48,49]. Targeting ferroptosis has been suggested to contribute to the prevention of the pathophysiological progression of several liver diseases, such as hemochromatosis, nonalcoholic steatohepatitis and ethanol-induced liver injury [50]. Ferroptosis inhibition also displays a protective effect in models of degenerative diseases, including Parkinson’s, Huntington’s and Alzheimer’s diseases [45,46,47,51,52]. Furthermore, the iron chelator deferiprone, which blocks ferroptosis, was reported to be beneficial in a randomized controlled clinical trial for Parkinson’s disease [53]. Interestingly, ferroptosis has also been reported to be involved in different types of human cancers, including breast, colorectal, lung, pancreatic, kidney and liver cancers [15,16,17,18]. Many recent studies have suggested that ferroptosis can act as a tumor suppressive mechanism, including studies on p53 and ferroptosis [15,16,17,18]. Furthermore, some cancer cells that are resistant to certain chemotherapeutic agents or targeted therapies have been shown to be sensitive to ferroptosis inducers [54,55,56,57]. However, currently, there is no specific biomarker to detect ferroptosis. Ferroptosis is usually confirmed by looking at whether cell death can be inhibited by specific ferroptosis inhibitors and by measuring lipid peroxides in cells [15,16,17,18]. Interestingly, a recent study reported that transferrin receptor 1 protein (TfR1) is a potential specific biomarker for ferroptosis. By screening ~4,750 antibodies generated from mice immunized with membranes from DLBCL cells undergoing ferroptosis, several anti-TfR1 antibodies, including 3F3 ferroptotic membrane antibody (3F3-FMA), were found to be able to detect ferroptosis in cell cultures and tissues [58].

Ferroptosis is different from apoptosis and other non-apoptotic cell death, such as autophagy and necrosis, at the morphological, biochemical and genetic levels. Morphologically, unlike apoptosis or necrosis, ferroptosis does not form apoptotic bodies or display chromatin condensation, cell shrinkage or rupture of the cell membrane [15,16,17,18]. Ferroptosis also does not form autophagosomes, which is typically observed in autophagy [15,16,17,18]. Biochemically, intracellular iron accumulation and peroxidation of polyunsaturated fatty acids (PUFAs) are required for ferroptosis [15,16,17,18]. While the detailed mechanism of how lipid peroxidation induces ferroptosis remains unclear, the iron and lipoxygenases are believed to be important contributors for lipid peroxidation and ferroptosis [59,60]. Ferroptosis can be inhibited by iron chelators (e.g., deferoxamine and desferrioxamine mesylate) and lipid peroxidation inhibitors (e.g., ferrostatin-1 and liproxstatin-1) but cannot be blocked by inhibitors of apoptosis, autophagy or necrosis [14,41,61]. Furthermore, iron overload resulting from the treatments of hemin, hemoglobin or iron chloride can induce ferroptosis in cells [15,16,17,62].

**Figure 2:**
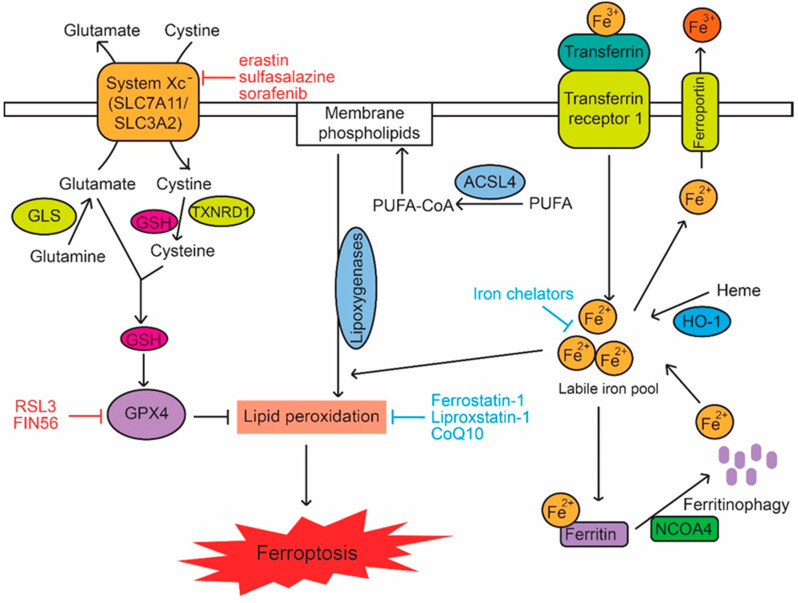
The signaling pathways in ferroptosis. The metabolic pathways involved in metabolism of cystine and glutamine, GSH, PUFAs and iron modulate lipid peroxidation in cells to regulate ferroptosis. CoQ10: coenzyme Q10; GLS: glutaminase; GSH: glutathione; PUFA: polyunsaturated fatty acid; PUFA-CoA: PUFA-coenzyme A.

Genetically, although the precise molecular mechanism of ferroptosis has not been fully understood, many different genes and pathways involved in regulation of iron metabolism, lipid synthesis and oxidative stress have been shown to regulate ferroptosis (Figure 2). For instance, GPX4, which is required for the efficient reduction of peroxidized phospholipids, has been shown to be a key upstream negative regulator of ferroptosis [41,63]. As a selenoperoxidase, GPX4 converts glutathione (GSH), an important intracellular antioxidant, into oxidized glutathione (GSSG) and reduces the cytotoxic lipid peroxides to the corresponding alcohols. Inhibition of GPX4 activity leads to the accumulation of lipid peroxides in cells [64]. It has been shown that compounds such as RSL3 and FIN56 can directly inhibit GPX4 activity to induce ferroptosis [54,63]. Acyl-CoA synthetase long chain family member 4 (ACSL4) has been demonstrated to be an additional critical regulator of ferroptosis [65,66]. ACSL4 is a fatty acid activating enzyme responsible for the esterification of PUFAs, especially long chain PUFAs (e.g., arachidonic acid and adrenic acid), to acyl-CoA in an ATP dependent manner. Knockdown or pharmacological inhibition of ACSL4 has been shown to inhibit ferroptosis, whereas overexpression of ACSL4 enhances sensitivity to ferroptosis in cells [65,66,67]. Furthermore, the cysteine-glutamate antiporter (system x_c_^-^), which is constituted by a substrate-specific subunit SLC7A11 and a regulatory subunit SLC3A2, has been shown to be critical for ferroptosis. System x_c_^-^ mediates the uptake of cystine, which is reduced to cysteine by GSH and/or thioredoxin reductase 1 (TXNRD1) and used for GSH biosynthesis [68]. A group of compounds, including erastin, sulfasalazine and sorafenib, have been shown to be the inhibitors of system x_c_^-^ -mediated cystine import, which can decrease the bioavailability of GSH to trigger ferroptosis [15,16,17,69]. Since elevated extracellular glutamate levels blocks the glutamate/cystine exchange of system x_c_^-^, enzymes that are involved in glutamate and glutamine metabolism (such as glutaminase GLS2) have been shown to be important regulators of ferroptosis [48,70]. Additionally, increasing the intracellular labile iron pool has also been shown to induce ferroptosis [15,16,17]. Many enzymes and proteins that affect the intracellular labile iron pool, including transferrin and transferrin receptor 1 (proteins that transport iron into cells), ferroportin (SLC11A3; an iron efflux pump and can oxidize Fe^2+^ to Fe^3+^), as well as heme oxygenase-1 (HO-1; an enzyme that catalyzes the degradation of cellular heme to liberate free iron) have been reported to regulate ferroptosis [48,62,71,72]. Ferritinophagy, a unique form of autophagy that degrades the iron-storage macromolecule ferritin (an iron binding protein) and nuclear receptor coactivator 4 (NCOA4; a ferritinophagy cargo receptor), contribute to ferroptosis through elevating labile iron pool and increasing oxygen radical formation in cells [15,16,17,73]. In addition to these above-mentioned proteins and enzymes directly involved in ferroptosis, some transcription factors have also been reported as regulators of ferroptosis. For instance, NRF2, a transcription factor that responds to oxidative stress, is involved in regulation of ferroptosis through transcriptionally inducing the expression of GPX4, SLC7A11 and HO-1 [74,75,76] (Figure 2). Interestingly, recent studies have revealed that p53 is also a key regulator of ferroptosis.

## 4. The Regulation of Ferroptosis by p53 and its Signaling Pathway

### 4.1. Wild-type p53 Induces Ferroptosis 

**Figure 3:**
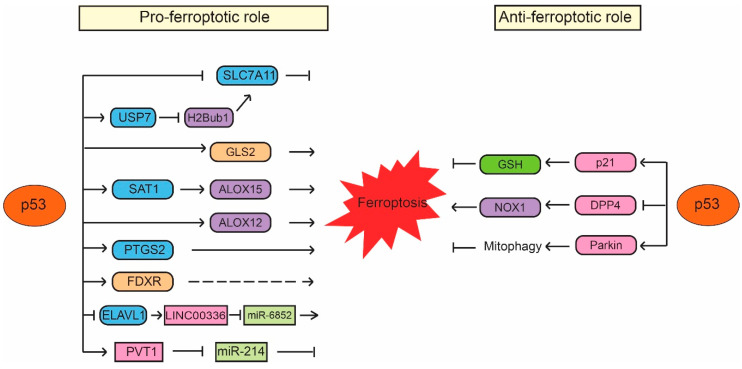
p53 regulates ferroptosis in a context-dependent manner. p53 promotes ferroptosis through its regulation of SLC7A11, GLS2, SAT1/ALOX15, ALOX12, PTGS2, FDXR, as well as the noncoding RNAs, such as LINC00336/miR-6852 and PVT1/miR-214. Meanwhile, p53 also inhibits ferroptosis through its regulation of p21, DPP4 and Parkin. The regulation of ferroptosis by p53 appears to be highly cell/tissue-type and stress signal specific.

p53 was first reported to sensitize cells to ferroptosis through transcriptionally repressing expression of SLC7A11 by Wei Gu’s group [77]. SLC7A11 is a direct p53 target; p53 binds to the p53-responsive element in the promoter region of SLC7A11 to repress its expression, leading to enhanced sensitivity of cancer cells to ferroptosis inducers, such as erastin [77]. Interestingly, p53^3KR^, an acetylation-defective p53 mutant containing mutations of three lysine residues (K117R, K161R and K162R) in the DNA-binding domain of p53, fails to induce cell cycle arrest, senescence and apoptosis but can effectively repress SLC7A11 expression and induce ferroptosis in response to ROS-induced stress [77]. Furthermore, p53-mediated ferroptosis contributes to embryonic development and the lethality induced by MDM2 knockout in mice [77]. SLC7A11 is frequently overexpressed in different types of human cancers, including colorectal, liver and kidney cancers [77,78,79]. Notably, ectopic expression of SLC7A11 inhibits ferroptosis and abolishes the tumor suppressive function of p53^3KR^ in xenograft tumor models [77]. The same group further showed that p53^4KR98^, an acetylation-defective p53 mutant with an additional lysine 98 (K98) mutation in p53^3KR^, fails to repress SLC711A expression or induce ferroptosis. Notably, p53^4KR98^ results in loss of tumor suppressive function of p53; compared with p53^3KR^ mice, the p53^4KR98^ mice are prone to tumor development [80]. These results provided one of the first lines of evidence that ferroptosis has tumor suppressive function and furthermore, the induction of ferroptosis by p53 contributes to the function of p53 in tumor suppression (Figure 3). Recently, monoubiquitination of histone H2B on lysine 120 (H2Bub1), an epigenetic mark for transcriptional activation, was reported to be involved in the regulation of SLC7A11 and ferroptosis [81]. H2Bub1 promotes SLC7A11 expression, whereas loss of H2Bub1 reduces SLC7A11 expression and leads to ferroptosis. Interestingly, p53 acts as a negative regulator of H2Bub1 by interacting with and promoting the nuclear translocation of USP7, a deubiquitinase that removes H2Bub1 from the regulatory region of the SLC7A11 gene, leading to decreased SLC7A11 expression. This p53-USP7-H2Bub1-SLC7A11 axis sensitizes cells to erastin-induced ferroptosis [81].

The low-molecular-weight polyamines, including putrescine, spermidine and spermine, are amino acid-derived polycationic alkylamines essential for cellular growth, proliferation and survival [82]. Their levels are tightly regulated by enzymes involved in polyamine metabolism, which is frequently dysregulated in different types of cancers, such as breast, lung and colorectal cancers [83]. SAT1 (Spermidine/spermine N1-acetyltransferase 1) is a rate-limiting enzyme that controls polyamine catabolism in cells [84]. Many stress signals, such as oxidative stress, inflammation and heat shock, activate SAT1 in cells. It has been reported that overexpression of SAT1 leads to the depletion of spermidine and spermine but the increased levels of putrescine, which induces mitochondrial apoptosis and inhibits cell proliferation [85,86]. Recently, SAT1 was shown to be a direct p53 target that can be induced by Nutlin-3 (a small molecule MDM2 inhibitor that activates p53 through disrupting the MDM2-p53 interaction) or the DNA-damaging agent doxorubicin in a p53-dependent manner in cells [87]. ROS-induced cell death in cells with SAT1 overexpression can be inhibited only by ferroptosis inhibitor ferrostatin-1 but not by inhibitors for other types of cell death, such as apoptosis inhibitor Z-VAD-FMK, necroptosis inhibitor necrostatin-1 or autophagy inhibitor 3-methyladenine [87]. SAT1 depletion inhibits p53-regulated ferroptosis in mouse embryonic fibroblasts (MEFs) from both wild-type p53 and p53^3KR^ mice [87]. Interestingly, SAT1 does not affect the levels and activities of SLC7A11 and GPX4. In contrast, the levels of ALOX15 (arachidonate 15-lipoxygenase), a member of the lipoxygenase family that oxygenates PUFAs and is responsible for oxidative stress-induced ferroptosis, are elevated upon SAT1 induction. Furthermore, ferroptosis induced by SAT1 and ROS can be effectively blocked by PD146176, an ALOX15-specific inhibitor, indicating that ALOX15 is a mediator of the p53-induced SAT1 expression and ferroptosis [87]. Additionally, ALOX12 (arachidonate 12-lipooxygenase), another member of the lipoxygenase family, was also identified as an important positive regulator for p53-mediated ferroptosis but not for GPX4 and ACSL4-mediated ferroptosis [88]. ALOX12 inactivation abrogates p53-mediated ferroptosis induced by ROS stress and abolishes the tumor suppressive function of p53 in xenograft tumor models. ALOX12 deficiency promotes tumorigenesis in Eμ-Myc lymphoma mouse models. Furthermore, tumor-associated ALOX12 missense mutations lose their lipoxygenase activity and ability to induce ferroptosis [88]. These results suggest that ALOX12 suppresses tumorigenesis through inducing ferroptosis, which contributes to the function of p53 in tumor suppression (Figure 3).

Ferroptosis is also modulated by glutamine metabolism. Upon the deprivation of amino acids, glutamine induces ferroptosis in a serum-dependent manner [48]. In glutamine catabolism, glutamine is firstly converted into glutamate by glutaminases (GLS1 and GLS2) and then further converted into α-ketoglutarate, an important substrate for the citric acid cycle (also named TCA cycle) in mitochondria [89]. GSL2, the liver-type glutaminase in mitochondria, has been identified as a direct transcriptional target of p53; p53 binds to the p53-responsive elements in the GLS2 gene to induce GLS2 transcription [90,91]. The induction of GLS2 mediates the p53 function in oxygen consumption and ATP generation in cells. Moreover, GLS2 promotes cellular antioxidant function by increasing GSH and NADH production in cells [90,91]. GLS2 displays a tumor suppressive function in liver and brain tumors, where GLS2 expression is frequently decreased [90,91,92,93,94]. Interestingly, GLS2 was reported to play a critical role in regulating ferroptosis; knockdown of GLS2 inhibits the serum-dependent ferroptosis induced by deprivation of amino acids [48]. In contrast, GLS1, a kidney-type glutaminase and a homology of GLS2, is not required for ferroptosis [48]. Further studies are necessary to understand whether GLS2 mediates p53-induced ferroptosis and why GLS2 but not GLS1 is required for ferroptosis given they both regulate glutaminolysis in cells. 

Cyclooxygenase-2 encoded by the PTGS2 gene is an enzyme that acts both as a peroxidase and a dioxygenase [95]. Cyclooxygenases catalyze lipid oxidation [96,97]. Studies have shown that PTGS2 expression levels are increased during the ferroptosis induced by RSL3, erastin or GPX4-deficiency in mice [42,63,98]. Interestingly, PTGS2 expression was reported to be upregulated by ferroptosis in a p53-dependent manner, which was only observed in p53^3KR/3KR^Mdm2^−/−^ embryos but not in p53^−/−^Mdm2^−/−^ embryos [77]. Currently, it is still unclear how p53 regulates PTGS2. Nevertheless, these results suggest that p53 promotes ferroptosis induced by RSL3 and erastin through upregulation of PTGS2 expression [77].

As mentioned above, iron metabolism is important for ferroptosis. It was reported that p53 regulates iron metabolism through ferredoxin reductase (FDXR), an enzyme that transfers electron from NADPH (nicotinamide adenine dinucleotide phosphate) to cytochrome P450 via ferredoxin in mitochondria [99]. FDXR is a p53 target gene and p53 induces transcriptional expression of FDXR [100]. At the same time, FDXR promotes p53 mRNA translation to increase p53 protein levels through iron regulatory protein 2 in cells [99]. This FDXR-p53 loop has been shown to be critical for tumor suppression through maintaining mitochondrial iron homeostasis; mice heterozygous in FDXR are prone to develop spontaneous tumors and display a short life span compared with wild-type mice [99]. Interestingly, RSL3- and erastin-induced ferroptosis can be suppressed by both FDXR deficiency and FDXR overexpression [99]. Although the underlying mechanism of this observation is unclear, this result suggests an additional potential mechanism of p53 in regulating ferroptosis through modulation of FDXR levels and iron metabolism in cells [99]. Further studies will help to reveal how FDXR and iron metabolism modulate p53-dependent ferroptosis (Figure 3).

In addition, recent studies have also reported that p53 induces ferroptosis through regulating noncoding RNAs. p53 has been reported to regulate expression of many noncoding RNAs, including microRNAs (miRNAs) and long noncoding RNAs (LncRNAs) and meanwhile, p53 is also regulated by many noncoding RNAs [101,102,103,104]. It was reported that p53 binds to the promoter region of RNA-binding protein ELAVL1 (ELAV-like RNA-binding protein 1) to repress the expression of ELAVL1, which binds to and stabilizes LncRNA LINC00336 in lung cancer cells [105]. LINC00336 inhibits ferroptosis by acting as an endogenous sponge of miRNA miR-6852, leading to the enhanced expression of cystathionine-β-synthase (CBS), which is involved in ferroptosis as a marker of transsulfuration pathway activity. Thus, p53 destabilizes LINC00336 and releases miR-6852, which in turn decreases the levels of CBS to promote ferroptosis [105]. Recently, LncRNA PVT1 was reported to induce ferroptosis through inhibition of miR-214-mediated downregulation of transferrin receptor 1 [106]. PVT1 can be induced by p53 and mediate the tumor suppressive function of p53 [107]. Therefore, it is possible that PVT1 mediates the function of p53 in promoting ferroptosis, contributing to the tumor suppressive function of p53. Since miR-214 can also repress p53, p53 may form a positive feedback loop with PVT1 to induce ferroptosis [106]. It was reported that the levels of PVT1 are upregulated and miR-214 levels are downregulated in the plasma from acute ischemic stroke patients [106], which suggests that p53-induced ferroptosis may contribute to ischemic stroke-induced tissue injuries in addition to tumor suppression (Figure 3).

### 4.2. Wild-Type p53 Inhibits Ferroptosis 

While many studies using different cultured cell systems and mouse models have shown the promoting effect of p53 on ferroptosis as summarized above, interestingly, some studies also reported that p53 can inhibit ferroptosis. p21 (also known as CDKN1A) was reported to mediate p53 function to delay cystine deprivation-induced ferroptosis in human fibrosarcoma HT-1080 cells [108]. p21 is a well-known p53 target gene that mediates the function of p53 in inducing cell cycle arrest and senescence in response to stress signals [1,2,3,4]. In HT-1080 cells, elevating p53 protein levels by treating cells with the MDM2 inhibitor nutlin-3 attenuates ferroptosis induced by erastin and knockout of p53 by CRISPR/Cas9 technology sensitizes cells to ferroptosis, suggesting a pro-survival role of p53 in ferroptosis [108]. Mechanistically, p53 induces p21 to increase the production of GSH, leading to the reduced accumulation of toxic lipid-ROS to inhibit ferroptosis (Figure 3). p21 exerts its functions in cell cycle arrest through binding to cyclin-dependent kinases (CDKs), including the CDK4/6 complex, and inhibiting their activities [109]. CDK4/6 inhibitors cannot block erastin-induced ferroptosis, which suggests that the inhibition of ferroptosis by p21 is independent of p21-mediated cell cycle arrest [108]. However, in non-cancerous human fetal lung IMR-90 fibroblast cells that express wild-type p53, nutlin-3 treatment increases p53 levels but does not affect the onset of erastin-induced ferroptosis, suggesting that the role of p53 in protection against ferroptosis is not universal [108]. Interestingly, a recent study reported that mutating the CDK binding region of p21 greatly abolishes the inhibitory effect of p21 on ferroptosis, while the PCNA binding-defective mutation of p21 only partially impairs the inhibitory effect of p21 on ferroptosis, which suggests that p21 alters sensitivity to ferroptosis by affecting CDK-mediated functions [110]. Further studies are needed to reveal the mechanism by which p21 regulates ferroptosis.

Recently, p53 was also reported to inhibit ferroptosis in colorectal cancer HCT116 and SW48 cells through binding to DPP4 protein (dipeptidyl peptidase-4; also known as T-cell activation antigen CD26), a multiple functional protease that plays an important role in mediating cell death [111]. The binding of p53 to DDP4 protein regulates the subcellular localization of DPP4 but not the protein levels of DPP4 [111]. In the absence of p53, DPP4 is localized on the plasma membrane, where it forms a complex with NADPH oxidase 1 (NOX1), a member of superoxide-generating NADPH oxidase protein family, to increase lipid peroxidation and ferroptosis. The binding of p53 to DPP4 sequesters DPP4 in a nuclear enzymatic inactive pool, leading to the NOX1 dissociation and decreased lipid peroxidation and ferroptosis [111] (Figure 3). Interestingly, depletion or inhibition of p53 only enhances ferroptosis induced by system x_c_^-^ inhibitors (e.g., erastin and sulfasalazine) but not ferroptosis induced by GPX4 inhibitors (e.g., RSL3 or FIN56) [111], suggesting that p53 regulates ferroptosis in a ferroptosis inducer-specific manner. Further, DPP4 inhibitors can block erastin-induced ferroptosis in p53-deficient colorectal cancer cells [111]. Interestingly, it was reported that while the expression of DPP4 is low in the normal adult colon, DPP4 is highly expressed in some colon cancers and cancer cell lines [112]. These findings suggest that the function of p53 in inhibiting ferroptosis might be specific for colorectal cancer cells with high DPP4 expression. 

Mitochondria have been reported to be involved in different programmed cell death processes, including apoptosis and mitophagy [113,114]. Recently, it was reported that mitochondria are also involved in ferroptosis [115]. Overexpression of Parkin, a protein involved in Parkinson’s disease and mitophagy, was shown to induce the mitophagy and inhibit cysteine deprivation-induced ferroptosis but not GPX4 inhibition-induced ferroptosis in human fibrosarcoma HT-1080 cells [115]. Mechanistically, cysteine deprivation results in the hyperpolarization of mitochondrial membrane potential and production of lipid peroxide, leading to ferroptosis. Blockage of mitochondrial electron transfer chain, TCA cycle or glutaminolysis attenuates mitochondrial membrane potential hyperpolarization, lipid peroxide accumulation and ferroptosis [115]. Interestingly, Parkin is a direct target gene of p53; p53 transcriptionally induce Parkin expression [116,117]. As a p53 target, Parkin mediates the function of p53 in regulating mitochondria respiration, oxygen consumption and anti-oxidant defense [116]. As a tumor suppressor, Parkin has been shown to be involved in metabolic regulation, including suppression of glycolysis and serine synthesis, contributing to the role of p53 in tumor suppression [116,118,119,120,121]. Currently, it remains unclear whether p53 is involved in cysteine deprivation-induced ferroptosis though regulation of Parkin expression. Taken together, p53 appears to regulate ferroptosis in a highly context-dependent manner, which leads to either positive or negative regulation of ferroptosis (Figure 3).

### 4.3. Ferroptosis Regulated by Mutp53, p53 Variants and p53 Family Proteins 

In addition to wild-type p53, missense mutp53 has also been reported to be involved in the regulation of ferroptosis. It has been known that NRF2 induces the expression of SLC7A11 to protect cancer cells from ferroptosis [122], whereas wild-type p53 transcriptionally represses SLC7A11 expression to induce ferroptosis [77]. Interestingly, mutp53 was reported to bind to NRF2 and inhibit its transcription activity, reducing the expression of SLC7A11 [69]. Tumors with missense mutp53 display decreased levels of SLC7A11 and increased sensitivity to ferroptosis [69]. Further, ectopic SLC7A11 expression in tumors with mutp53 expression promotes resistance of tumors to ferroptosis-inducing drugs (e.g., sulfasalazine), which suggests that mutp53 sensitizes the cancer cells to ferroptosis through repression of SLC7A11 expression [69]. Similar effects of mutp53 were also observed in human colorectal cancer cells; cells expressing mutp53 are more sensitive to erastin-induced ferroptosis than cells expressing wild-type p53 and ectopic expression of R175H mutp53, a GOF mutp53 hotspot mutation, restores the sensitivity to erastin in both HCT116 and SW48 cells [111]. Given that GOF mutp53 often accumulates to very high levels in cancer cells and renders cancer cells resistant to chemotherapy and radiation therapy as a GOF activity, these findings suggest that some cancers expressing GOF mutp53 may be more sensitive to ferroptosis inducers, which can be a potential therapeutic strategy for these cancers. It has been reported that different mutp53 can differ in the magnitudes of their GOF activities and display GOF activities in a cell- or tissue-specific manner [6,123,124]. Therefore, it will be crucial to investigate whether sensitizing cancer cells to ferroptosis inducers is a general phenomenon for different GOF mutp53 in different types of cancers.

Some common single nucleotide polymorphisms (SNPs) in the p53 gene have been known to affect the p53 transcriptional activity and functions in tumor suppression, metabolism, maternal reproduction and aging [27,31,125,126]. The Pro47Ser variant (hereafter S47) is the second most common SNP in the p53 coding region (after Pro72Arg). In a humanized p53 knock-in mouse model of S47 variant, mice expressing homozygous or heterozygous S47 p53 variant are more susceptible to spontaneous cancers of diverse histological types (particularly liver cancer) compared with wild-type mice [127]. Similar to p53-/- MEFs, MEFs with S47 p53 variant display a high GSH/GSSG ratio and low-molecular-weight thiols coenzyme A level and are less sensitive to ferroptosis compared with wild-type MEFs [128]. The S47 p53 variant shows an impaired ability to transcriptionally induce a subset of p53 target genes, including two well-known genes involved in metabolism, *GLS2* and *SCO2,* suggesting that the defect in metabolic regulation may contribute to the reduced ferroptosis and the tumor-prone phenotype observed in S47 mice [127]. Notably, further studies showed that the ferroptotic defect in S47 p53 variant results in iron accumulation in macrophages, which alters macrophage cytokine profiles and leads to the higher level and activity of arginase and decreased activity of nitric oxide synthase [129]. S47 macrophages have decreased liver X receptor (LXR) activation, inflammation and antibacterial defense. S47 mice display more productive intracellular bacterial infections but are protective against malarial toxin hemozoin. Furthermore, iron chelators and LXR agonists can improve the response of S47 mice to bacterial infection [129]. These results suggest that p53-mediated ferroptosis plays an important role in regulation of iron accumulation to modulate macrophage functions and host immune responses [129].

p63 and p73 are two p53 family members, which are homologous to p53 and function as transcription factors like p53 [27,130,131]. Both p63 and p73 can be transcribed from different promotors resulting in the expression of isoforms with different N-terminal domains, including transcription activation domain (TA) and ΔN (amino-truncated) isoforms. Studies have suggested that the TA isoforms can function as tumor suppressors and induce the expression of canonical p53 target genes, whereas the ΔN isoforms can function as oncogenes and antagonize p53, TAp63 and TAp73 by inhibiting their transcriptional activities [27,130,131]. Interestingly, ΔNp63α was reported to inhibit both erastin- and RSL3-induced ferroptosis independently of p53 [132]. ΔNp63α transcriptionally regulates GSH biogenesis, utilization and regeneration, leading to the reduction of lipid peroxidation. Furthermore, ΔNp63α upregulates SLC7A11 expression but is unable to inhibit p53-mediated transcriptional repression of SLC7A11. As a result, ΔNp63α induces the expression of SLC7A11 in p53-/- MEFs but not in wild-type MEFs where p53 represses the expression of SLC7A11 [132]. Nevertheless, ΔNp63α protects both wild-type and p53-/- MEFs from ferroptosis induced by erastin and RSL3, indicating that the regulation of GSH homeostasis by ΔNp63α is sufficient to inhibit ferroptosis [132]. Currently, it remains unclear whether p73 is also involved in the regulation of ferroptosis. 

### 4.4. Ferroptosis Regulated by MDM2 and MDM4 Independently of p53

Although MDM2 and MDM4 are critical negative regulators of p53, many studies have shown that MDM2 and MDM4 also exert oncogenic effects independently of their activities to inhibit p53 function [37,38]. Interestingly, a recent study reported that MDM2 and MDM4 also display a p53-independent function to promote ferroptosis [133]. Both inhibitors of MDM2 (MEL23) and MDM4 (NCS207895) protect cells from ferroptosis induced by erastin and RSL3 through enhancing the levels of ferroptosis suppressor protein 1 (FSP1), an enzyme that reduces coenzyme Q10, an endogenous lipophilic antioxidant, to ubiquinol, which in turn enhances the levels of coenzyme Q10 [133]. Further, the MDM2-MDM4 complex reprograms lipid metabolism by altering the activity of PPARα, leading to enhanced lipid peroxidation and ferroptosis independently of p53 [133]. These results suggest that cancers with MDM2/MDM4 amplification and/or overexpression might be more sensitive to ferroptosis inducers, which can be tested as a potential therapeutic strategy for these cancers. 

### 4.5. p53 Regulation in Response to Ferroptosis Stimuli and Inhibition

As a stress sensor, p53 responds to many different types of stress signals and initiate its transcription program to regulate different stress responses [1,2,3,4]. While many studies have shown that p53 is involved in the regulation of ferroptosis in a highly context-dependent manner, an interesting question raised is how p53 responds to different ferroptosis inducers and inhibitors to regulate ferroptosis. Some studies have shown that p53 can be activated by ferroptosis inducers or inhibitors. For example, it was reported that erastin can induce p53 protein levels and increase p53 transcriptional activity towards its target genes, including p21 and Bax, in human lung cancer A549 cells [134]. Treating cells with ROS scavenger N-acetyl-1-cysteine (NAC) abolishes the effect of erastin on p53 levels, which suggests that enhancing the ROS levels is a mechanism contributing to p53 activation by erastin [134]. Compound D13, a derivative of the triterpene saponin natural compound Albiziabioside A, was reported to induce ferroptosis through suppressing GPX4 expression in human HCT116 colorectal cancer cells [135]. D13 displays an anti-tumor effect in xenograft tumors formed by HCT116 cells. Furthermore, D13 activates p53 and displays a p53-dependent effect on ferroptosis [135]. Interestingly, iron chelators, including desferrioxamine, have been reported to activate p53 [136,137,138]. HIF-1α may play an important role in mediating the induction of p53 by iron chelating agents. It has been reported that iron deprivation caused by iron chelating agents results in the activation of HIF1α [139,140] and HIF-1α stabilizes p53 through inhibiting the ubiquitination and nuclear export of p53 [141,142]. A recent study reported that the iron polyporphyrin heme inhibits p53 function by binding to p53 and leading to nuclear export and degradation of p53 [143]. Therefore, iron deprivation induced by desferrioxamine activates p53 and suppresses growth of HCT116 xenograft tumors in a p53-dependent manner. However, it is unclear how ferroptosis contributes to this the tumor suppressive effect of desferrioxamine and how p53 activation by desferrioxamine contributes to ferroptosis in this study [143]. Ferroptosis inhibitors, including ferrostatins, liproxstatins and iron chelators, have been reported to display protective effects on ischemic injuries and neurodegenerative diseases [41,43,45,46,47,48,49,51,52]. The iron chelator deferiprone was reported to display a beneficial effect on Parkinson’s disease patients [53]. Interestingly, p53 has been reported to be involved in ischemic injuries and neurodegenerative diseases [1,2,3,4]. Currently, it remains largely unknown how p53 responds to these ferroptosis inhibitors and how p53 is involved in ferroptosis in these conditions and models. Given the critical roles of p53 and ferroptosis in the development and therapy of human diseases, including cancer, ischemic injuries and neurodegenerative diseases, future studies are needed to further elucidate how p53 responds to different ferroptosis inducers and inhibitors to regulate ferroptosis in a cell-, tissue-type and disease-specific manner. 

## 5. Summary and Perspectives

As a new form of programmed cell death, although remarkable progress has been made on the research on ferroptosis since its discovery in 2012, our current understanding of the molecular mechanism of ferroptosis, the role of ferroptosis in biological and pathological processes, as well as its application in treatments of cancer and other diseases is still limited. As summarized above, p53 has been shown to play an important role in regulation of ferroptosis. Notably, the original concept that ferroptosis can function as a tumor suppressive mechanism came from the studies showing the promoting effect of p53 on ferroptosis in mouse models [77,80]. However, there are many important and urgent questions regarding the regulation of ferroptosis by p53 have not been addressed yet. For example, like some other functions of p53, the function of p53 in regulation of ferroptosis appears to be highly context-dependent; p53 has been shown to be able to both promote and suppress ferroptosis. Further studies are warranted to understand whether this context dependence is caused by cell and tissue specificity, stress signal specificity and/or ferroptosis inducer specificity and to identify the signaling pathways and molecular mechanisms contributing to this context dependence. Furthermore, given that ferroptosis is heavily regulated by metabolic pathways and affected by nutrition availability in the cellular environment and that many studies on p53 regulation of ferroptosis are based on in vitro cell culture systems, it is unclear whether some of these observations made using in vitro cell culture systems can be recapitulated by using in vivo animal models. More studies using animal models will help to elucidate whether and how p53 regulates ferroptosis in cell, tissue and organ type-specific, developmental stage-specific and ferroptosis inducer-specific manners. Considering that the key role of p53 in tumor suppression, another interesting question is that how to reconcile the observed opposite effects of p53 in both promoting and suppressing ferroptosis. An interesting hypothesis is that ferroptosis might function as a double-edged sword that can both inhibit and promote cancer progression under different circumstances [16]. A good example is autophagy, which has dual functions in both tumor suppression and promotion [144,145]. If this is the case, p53 might inhibit ferroptosis to suppress cancer progression under certain conditions. Then it will be crucial to identify the signaling pathways and mechanisms that determine whether p53-regulated ferroptosis inhibits or promotes cancer progression. In addition to tumor suppression, p53 has been demonstrated to regulate many other physiological and pathological processes, such as metabolism, immune response, tissue ischemia/reperfusion injuries and neurodegeneration. Interestingly, recent studies have also revealed that ferroptosis plays an important role in regulating metabolism, immune response, neurodegeneration and tissue ischemia/reperfusion injuries. It is possible that p53 is involved in these processes in a tissue- and stress-dependent manner through promoting or inhibiting ferroptosis. It is worth noting that p53-regulated ferroptosis has been shown to play an important role in differentially regulating host immune responses to bacterial and malarial infections through modulating macrophage functions [129]. While many studies on the regulation of ferroptosis by p53 have focused on the tumor suppressive function of p53, it is crucial to determine the contribution of ferroptosis to the role of p53 in metabolism, immune response, neurodegeneration and tissue ischemia/reperfusion injuries. Future studies will shed further light on the role and mechanism of ferroptosis and p53-regulated ferroptosis in different biological and pathological processes, which will lead to new and effective therapeutic strategies for cancers and other diseases.
